# Spatial Access to Continuous Maternal and Perinatal Health Care Services in Low-Resource Settings: Cross-Sectional Study

**DOI:** 10.2196/49367

**Published:** 2024-07-18

**Authors:** Qin Li, Elsa Kanduma, Isaías Ramiro, Dong (Roman) Xu, Rosa Marlene Manjate Cuco, Eusebio Chaquisse, Yili Yang, Xiuli Wang, Jay Pan

**Affiliations:** 1 HEOA Group West China School of Public Health, West China Fourth Hospital Sichuan University Chengdu China; 2 Institute for Healthy Cities, West China Research Center for Rural Health Development Sichuan University Chengdu China; 3 Comité para a Saúde de Moçambique Maputo City Mozambique; 4 Acacia Lab for Implementation Science, Center for World Health Organization Studies School of Health Management Southern Medical University Guangzhou China; 5 SMU Institute for Global Health Dermatology Hospital Southern Medical University Guangzhou China; 6 Mozambique Ministry of Health and Faculty of Medicine Eduardo Mondlane University Maputo City Mozambique; 7 China Center for South Asian Studies Sichuan University Chengdu China

**Keywords:** continuous maternal and perinatal health care services, sub-Saharan Africa, SSA, spatial access, resource allocation, low-resource settings

## Abstract

**Background:**

Maternal and perinatal health are fundamental to human development. However, in low-resource settings such as sub-Saharan Africa (SSA), significant challenges persist in reducing maternal, newborn, and child mortality. To achieve the targets of the sustainable development goal 3 (SDG3) and universal health coverage (UHC), improving access to continuous maternal and perinatal health care services (CMPHS) has been addressed as a critical strategy.

**Objective:**

This study aims to provide a widely applicable procedure to illuminate the current challenges in ensuring access to CMPHS for women of reproductive age. The findings are intended to inform targeted recommendations for prioritizing resource allocation and policy making in low-resource settings.

**Methods:**

In accordance with the World Health Organization guidelines and existing literature, and taking into account the local context of CMPHS delivery to women of reproductive age in Mozambique, we first proposed the identification of CMPHS as the continuum of 3 independent service packages, namely antenatal care (ANC), institutional delivery (ID), and postnatal care (PNC). Then, we used the nearest-neighbor method (NNM) to assess spatial access to each of the 3 service packages. Lastly, we carried out an overlap analysis to identify 8 types of resource-shortage zones.

**Results:**

The median shortest travel times for women of reproductive age to access ANC, ID, and PNC were 2.38 (IQR 1.38-3.89) hours, 3.69 (IQR 1.87-5.82) hours, and 4.16 (IQR 2.48-6.67) hours, respectively. Spatial barriers for women of reproductive age accessing ANC, ID, and PNC demonstrated large variations both among and within regions. Maputo City showed the shortest travel time and the best equity within the regions (0.46, IQR 0.26-0.69 hours; 0.74, IQR 0.47-1.04 hours; and 1.34, IQR 0.83-1.85 hours, respectively), while the provinces of Niassa (4.07, IQR 2.41-6.63 hours; 18.20, IQR 11.67-24.65 hours; and 7.69, IQR 4.74-13.05 hours, respectively) and Inhambane (2.69, IQR 1.49-3.91 hours; 4.43, IQR 2.37-7.16 hours; and 10.76, IQR 7.73-13.66 hours, respectively) lagged behind significantly in both aspects. In general, more than 51% of the women of reproductive age, residing in 83.25% of Mozambique’s land area, were unable to access any service package of CMPHS in time (within 2 hours), while only about 21%, living in 2.69% of Mozambique’s land area, including Maputo, could access timely CMPHS.

**Conclusions:**

The spatial accessibility and equity of CMPHS in Mozambique present significant challenges in achieving SDG3 and UHC, especially in the Inhambane and Niassa regions. For Inhambane, policy makers should prioritize the implementation of a decentralization allocation strategy to increase coverage and equity through upgrading existing health care facilities. For Niassa, the cultivation of well-trained midwives who can provide door-to-door ANC and PNC at home should be prioritized, with an emphasis on strengthening communities’ engagement. The proposed 2-step procedure should be implemented in other low-resource settings to promote the achievement of SDG3.

## Introduction

Access to continuous maternal and perinatal health care services (CMPHS) has been proposed by the World Health Organization (WHO) as an essential strategy to improve maternal and child health [1]. Instead of focusing on single interventions, CMPHS emphasizes packages of interventions delivered throughout the lifecycle at all levels of health care facilities in the health delivery system [2]. The provision of CMPHS is also a priority for achieving universal health coverage (UHC) and is listed as a key indicator for monitoring the progress of maternal, newborn, and child outcomes [3]. It has been widely recognized that CMPHS can be understood as the integration of 3 service delivery packages: antenatal care (ANC), institutional delivery (ID), and postnatal care (PNC) [4]. ANC facilitates the prompt detection and management of complications among pregnant women [5]. Research suggests that even a 7% increase in ANC coverage might save 160,000 newborn lives annually in Africa [6]. ID ensures appropriate equipment and supplies are available on site or through immediate referral to a higher-level facility if necessary [7]. As suggested by the literature, perinatal mortality is 21% lower for facility-based deliveries compared to home deliveries. In the best settings, up to 14 perinatal deaths might be averted per 1000 births if the obstetric deliveries occurred at facilities instead of at homes [8]. PNC provides great opportunities to check for dangerous signs and symptoms associated with poor health outcomes in babies [9]. The importance of PNC has been documented, with an estimation that if PNC rates reach 90% in sub-Saharan Africa (SSA), neonatal deaths could be averted by 10%-27% [10].

Mozambique represents a low-resource setting of the type prevalent in the SSA region, where the world’s highest maternal and neonatal mortality persists as a critical issue (533 maternal deaths per 100,000 live births vs 211 per 100,000 worldwide and 27 neonatal deaths per 1000 live births vs 18 per 1000 worldwide). Timely provision of CMPHS could prevent a significant proportion of these deaths [11-13]. Several recent studies have examined health care accessibility in low-resource settings, including emergency obstetric and newborn care, skilled birth attendance and caesarean delivery services, and primary health care [14-18]. However, efforts to assess the spatial accessibility of CMPHS remain insufficient [19] despite widespread recognition that the effectiveness of the 3 service packages is greatly influenced by health care services achieved in the previous stages. Moreover, there is a lack of targeted suggestions on resource investment and policy making in this area.

Among various barriers preventing women of reproductive age from obtaining timely access to CMPHS, spatial accessibility (geographic proximity) has become one of the primary barriers, particularly in low-resource settings [20]. As a crucial aspect of accessibility, use, and the provision of health care services to the population group in need [21], assessment of spatial accessibility has been carried out worldwide and been incorporated into policy making in many countries. However, there is a paucity of studies assessing the spatial accessibility of CMPHS by integrating multiple indicators into 3 separate service packages in low-resource settings.

As the deadline for sustainable development goal 3 (SDG3) approaches, and the fundamental need for maternal and prenatal health care persists, the efficient allocation of limited health care resources, especially in low-resource settings, is of great importance. Mozambique, one of the world’s poorest and most underdeveloped countries (with a United Nations Human Development Index of 0.446 in 2021), faces significant challenges in implementing SDG3, particularly concerning CMPHS indicators [22]. This study uses Mozambique as a case study, aiming to provide a widely applicable procedure for assessing the spatial access to CMPHS and to offer evidence-based suggestions for policy makers that will guide the prioritization of health resource allocation to improve CMPHS accessibility and maternal, newborn, and child health outcomes in low-resource settings.

## Methods

### Study Area

Mozambique, located on the southeast coast of Africa, is divided into 11 provinces, including 1 provincial city (Multimedia Appendix 1, Figure S1A). According to data announced by the General Population and Housing Census 2017 in Mozambique [23], the estimated total population was 30.07 million, with the majority living in rural areas and generally sparsely and unevenly distributed (Multimedia Appendix 1, Figure S1B). Nearly 80% of people living in poverty live in rural areas distant from basic public facilities [24]. The country’s overall economy remains extremely underdeveloped (gross domestic product <US $1.00/person/day); the highest average consumption level in Maputo City is 6.9 times higher than the lowest, in Zambezia province (Multimedia Appendix 1, Figure S1C) [25].

According to a report from the Mozambique Ministry of Health, the country’s maternal mortality rate was as high as 451 deaths per 100,000 live births, and the infant mortality rate was 67.4 deaths per 1000 live births in 2020 [26]. Factors such as long travel distances from residential locations to health care facilities, lack of available transportation, and poorly constructed transportation networks contribute to significant service delays for women of reproductive age accessing CMPHS [15,27].

### Data Sources

Data on 2 perspectives were needed for identifying indicators reflective of the delivery of the 3 service packages in Mozambique for calculating spatial accessibility. Following the existing literature and considering both representativeness and implementability, 2 categories of data were used for indicator identification and 3 categories of data were used to facilitate the analysis of spatial accessibility, including supply side data (health care facility data), demand side data (distribution data for women of reproductive age), and environmental data (for calculating geospatial barriers) [28-30]. The data sources are shown in detail in Table 1.

**Table 1:** Description and sources of data use in the 2 steps of the proposed procedure.

Perspectives	Data type	Data description	Data sources
Identifying indicators representing 3 service packages of CMPHS^a^ in Mozambique	For representativeness: global guidelines	Guidelines for essential practice in maternal, newborn, and child health and pregnancy, childbirth, postpartum, and newborn care	World Health Organization guidelines [31,32]
	For implementability: Mozambique-specific data	Continuous maternal and perinatal health care services (antenatal care, institutional delivery, and postnatal care)	The questionnaire of the National Survey on Infrastructure, Equipment, Human Resources and Health Services 2018
Calculating spatial accessibility of CMPHS in Mozambique	Supply side: health care facility data	Geographic coordinates (longitude, latitude) of all health care facilities	Public health facilities in sub-Saharan Africa; Figshare data set [33] and Google Maps
		Address of all health care facilities	The questionnaire of the National Survey on Infrastructure, Equipment, Human Resources and Health Services 2018
	Demand side: population data	Data on number of women of reproductive age at the provincial level (2018)	*Statistical Yearbook 2018 of Mozambique* [34]
		Raster data on spatial distribution of pregnancies at 1×1 km^2^ resolution (2015)	WorldPop provides open geospatial data on distribution of pregnancies in Mozambique [35]
	Environmental data	Administrative boundaries	OpenStreetMap [36]

^a^CMPHS: continuous maternal and perinatal health care services.

### Data Pretreatment and Analysis

**Figure 1:**
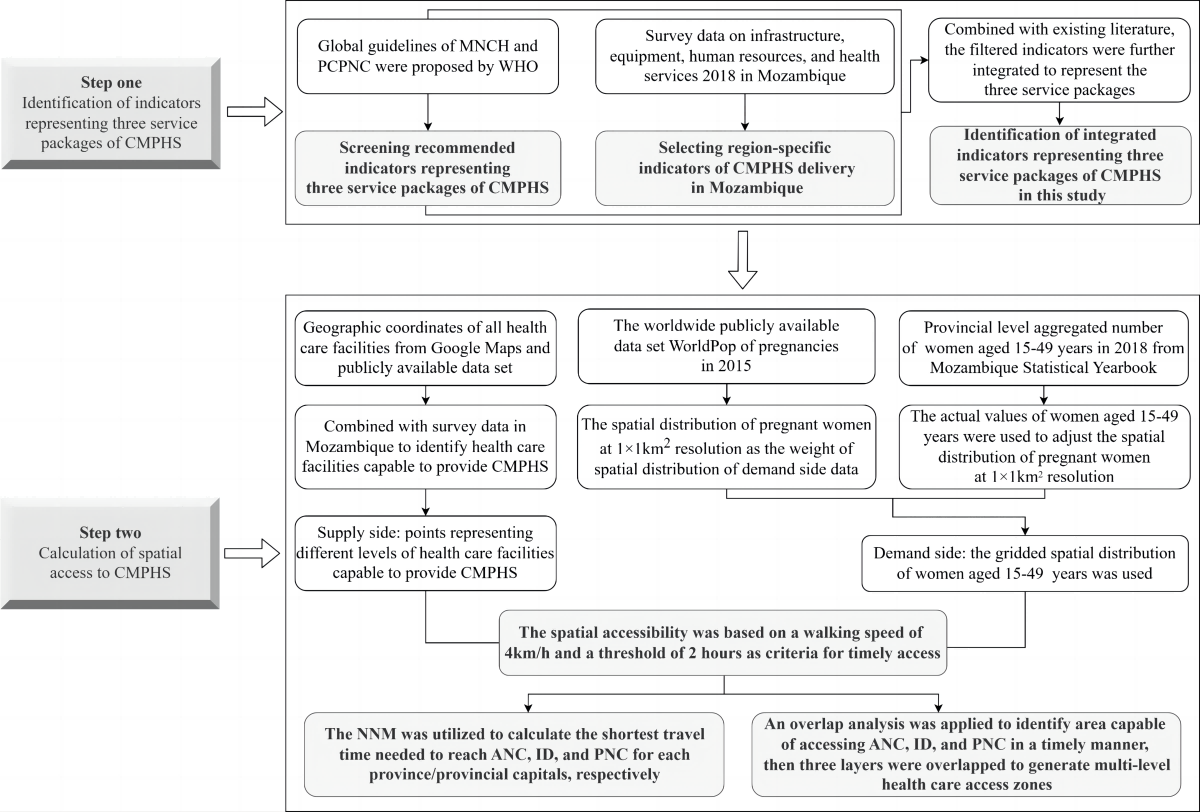
Flowchart of the proposed 2-step procedure, including the main process and data used in each process. ANC: antenatal care; CMPHS: continuous maternal and perinatal health care services; ID: institutional delivery; MNCH: maternal, newborn, and child health; PCPNC: pregnancy, childbirth, postpartum and newborn care; PNC: postnatal care; WHO: World Health Organization.

A detailed flowchart describing the 2-step procedure is shown in Figure 1.

#### Step 1: Identification of Indicators Representing the 3 Service Packages of CMPHS

The selection of indicators was based on 2 main principles. The first principle was accordance with maternal, newborn, and child health (MNCH) guidelines, as well as the pregnancy, childbirth, postpartum, and newborn care (PCPNC) guidelines proposed by the WHO. The second principle was to fully reflect the region-specific characteristics of CMPHS delivery in the study area.

In adhering to the 2 main principles, recommended and available indicators were first filtered to identify a group of separate indicators from each individual service package. The available indicators were extracted from Mozambique’s Survey on Infrastructure, Equipment, Human Resources and Health Services 2018 (Multimedia Appendix 1, Table S1) [37,38]. Then, with consideration of both avoiding fragmented services and ensuring the quality of CMPHS, as well as extensive reference to existing literature, [39], the filtered indicators were further screened and integrated to the 3 service packages. ANC includes 7 integrated indicators, ID includes 8 integrated indicators, and PNC includes 10 integrated indicators (Multimedia Appendix 1, Table S2).

#### Step 2: Calculation of Spatial Access to CMPHS (Data Pretreatment)

For the supply side data, 3 levels of health care facilities were included, consisting of Mozambique’s health care delivery system: primary (urban and rural health centers and community health posts), secondary (rural, district, and general hospitals), and tertiary (central and provincial, specialized, and military hospitals). Combined with results from step 1, the capability of delivering the 3 service packages of CMPHS was identified for every health care facility.

For demand side data, gridded estimates of pregnancies were obtained at 1×1 km^2^ spatial resolution from the WorldPop database for 2015, which served to weight the spatial distribution of the demand side data. Then, data on the nationally announced provincial-level aggregated number of women aged 15-49 years in Mozambique was used to adjust the spatial distribution of pregnant women at a resolution of 1×1 km^2^ to generate a more accurate and up-to-date demand distribution, as process generally used in related research [40-42].

The following formula was used to calculate the spatial distribution of women of reproductive age at 1×1 km^2^ resolution [40], in which *P_ij_* is the number of women of reproductive age of grid *i* in the province or provincial capital *j*, and *G_ij_* is the corresponding WorldPop value for pregnancy density of grid *i*. *G_j_* is the sum of the grid values for WorldPop in province or provincial capital *j*, and *T_j_* is the actual number of women of reproductive age in province or provincial capital *j* from the *Statistical Yearbook 2018 of Mozambique* [23]:

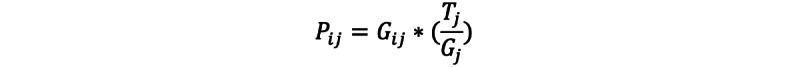



### Analysis of Spatial Accessibility (Establishment of Geo-Database)

ArcGIS (version 10.5; Esri, Inc) was used to calculate spatial access to CMPHS in this study. A geodatabase incorporating supply side data, demand side data, and administrative boundary data was first generated [43].

### Calculation of the Shortest Travel Time Needed to Reach ANC, ID, and PNC

The nearest-neighbor method (NMM) was used to calculate the shortest travel time necessary to access ANC, ID, and PNC [44]. For each grid of women of reproductive age, only the nearest health care facility was considered [41]. The travel mode of walking at a speed of 4 km/h for spatial accessibility assessment and a threshold of 2 hours were used as criteria for timely access (Multimedia Appendix 1, Table S3) [5]. Additionally, the travel time along the straight-line distance was used to compare the travel time criterion [18,45]. The shortest travel time needed to reach ANC, ID, and PNC was calculated separately, and the average travel time was calculated for each province or provincial capital.

### Overlap Analysis of Spatial Coverage of CMPHS

An overlap analysis was applied to identify multilevel health care access zones. On the basis of identifying areas able to access ANC, ID, and PNC in a timely manner (ie, within a 2 h threshold), 3 layers representing timely access to ANC, ID, and PNC, respectively, were overlapped to generate 8 types of multilevel health care access zones. Then, percentages for area and women of reproductive age covered within each type of multilevel health care access zone were calculated.

### Ethical Considerations

The study was approved by the Swiss Confederation, represented by the Swiss Federal Department of Foreign Affairs, acting through the Swiss Agency for Development and Cooperation Global Cooperation Department and Southern Medical University of the People’s Republic of China (81067392). Core data used in this study were derived from the National Survey on Infrastructure, Equipment, Human Resources and Health Services 2018, which was collected between February and April 2018 through a cross-sectional survey of health care facilities. The survey included a census of all health facilities in the country and collected data through interviews with key informants at health care facilities. The core standard Service Availability and Readiness Assessment (SARA) questionnaire of the WHO was used and adapted to the Mozambique context [37,38]. The SARA questionnaire and linked protocols were granted approval by Mozambique’s Ministry of Health. More information about the primary data collection procedures is available in the Mozambique SARA 2018 report [38]. Permission to use the data was obtained from Mozambique’s Ministry of Health by coauthors from Mozambique. Other data, including population distribution and administrative boundaries, were extracted from publicly available data sets. No human participants or animals were involved in the study.

## Results

### Identification and Coverage of Indicators Representing CMPHS

The identified indicators for the 3 service packages and the percentage of health care facilities capable of providing each service (indicators) are shown in Table 2. Among all health care facilities (N=1542), 784 were able to provide ANC, 365 were able to provide ID, and 299 were able to provide PNC.

Most health care facilities within the study area managed to cover all services listed in each individual service package, with some indicators having fairly good penetration. The provision of intermittent preventive treatment in pregnancy for malaria, a component of the ANC service package, was offered in 1408 (91.31%) health care facilities. Conversely, some indicators showed suboptimal coverage; for example, emergency obstetric care was available in only 504 (32.68%) health care facilities and injectable antibiotics intended for neonatal sepsis were available in just 766 (49.68%) health care facilities (Table 2).

**Table 2:** Identified indicators of the 3 service packages and the percentage of health care facilities able to provide each service (indicators) based on global guidelines for maternal, newborn, and child health, as well as pregnancy, childbirth, postpartum, and newborn care proposed by the World Health Organization and data extracted from the National Survey on Infrastructure, Equipment, Human Resources and Health Services 2018.

Phases and key indicators		Health care facilities providing antenatal care, institutional delivery, and postnatal care (N=1542), n (%)
**Antenatal care (n=7 indicators; n=784 health care facilities)**		
	Iron supplementation	1070 (69.39)
	Folic acid supplementation	1035 (67.12)
	Tetanus toxoid vaccination	1364 (88.46)
	Monitoring for hypertensive disorder of pregnancy	1198 (77.69)
	IPTp^a^ for malaria	1408 (91.31)
	HIV counseling and testing for HIV-positive pregnant women	1369 (88.78)
	Antiviral treatment for HIV-positive pregnant women	1347 (87.35)
**Institutional delivery** **(n=8 indicators; n=365 health care facilities)**		
	Monitoring of labor with partograph	1273 (82.56)
	Parenteral administration of oxytocin	1243 (80.61)
	Assisted vaginal delivery	1125 (72.96)
	Manual removal of placenta	1103 (71.53)
	Antibiotics for preterm	849 (55.06)
	Blank partograph	1204 (78.08)
	Parenteral administration of magnesium sulfate	1079 (69.97)
	Emergency obstetric care	504 (32.68)
**Postnatal care (n=10 indicators; n=299 health care facilities)**		
	Immediate and exclusive breastfeeding	1313 (85.15)
	Thermal protection	1293 (83.85)
	Hygenic cord care	1307 (84.76)
	Neonatal resuscitation	1122 (72.76)
	Kangaroo mother care	1094 (70.95)
	Injectable antibiotics for neonatal sepsis	766 (49.68)
	HIV counseling and testing for infants born to HIV-positive women	1351 (87.61)
	ARV^b^ prophylaxis to newborns of HIV-positive pregnant women	1345 (87.22)
	HIV-positive infant and young child feeding counseling	1367 (88.65)
	BCG^c^ vaccine	886 (57.46)

^a^IPTp: intermittent preventive treatment in pregnancy.

^b^ARV: AIDS-related virus.

^c^BCG: bacillus Calmette-Guérin.

### Distribution of Health Care Facilities Providing CMPHS

There were 1542 health care facilities in total across the study area, among which were 1490 primary-level health care facilities (urban and rural health centers or community health posts), 43 secondary-level health care facilities (rural, district, and general hospitals), and 9 tertiary-level health care facilities (central and provincial hospitals or specialized and military hospitals). Among the primary-level facilities, 51.48% (767/1490), 21.34% (318/1490), and 18.46% (275/1490) were able to provide ANC, ID, and PNC, respectively. For the secondary facilities, the respective percentages were 37% (16/43), 91% (39/43), and 23% (17/43). For the tertiary-level health care facilities, the respective percentages were 11% (1/9), 89% (8/9), and 78% (7/9).

**Figure 2:**
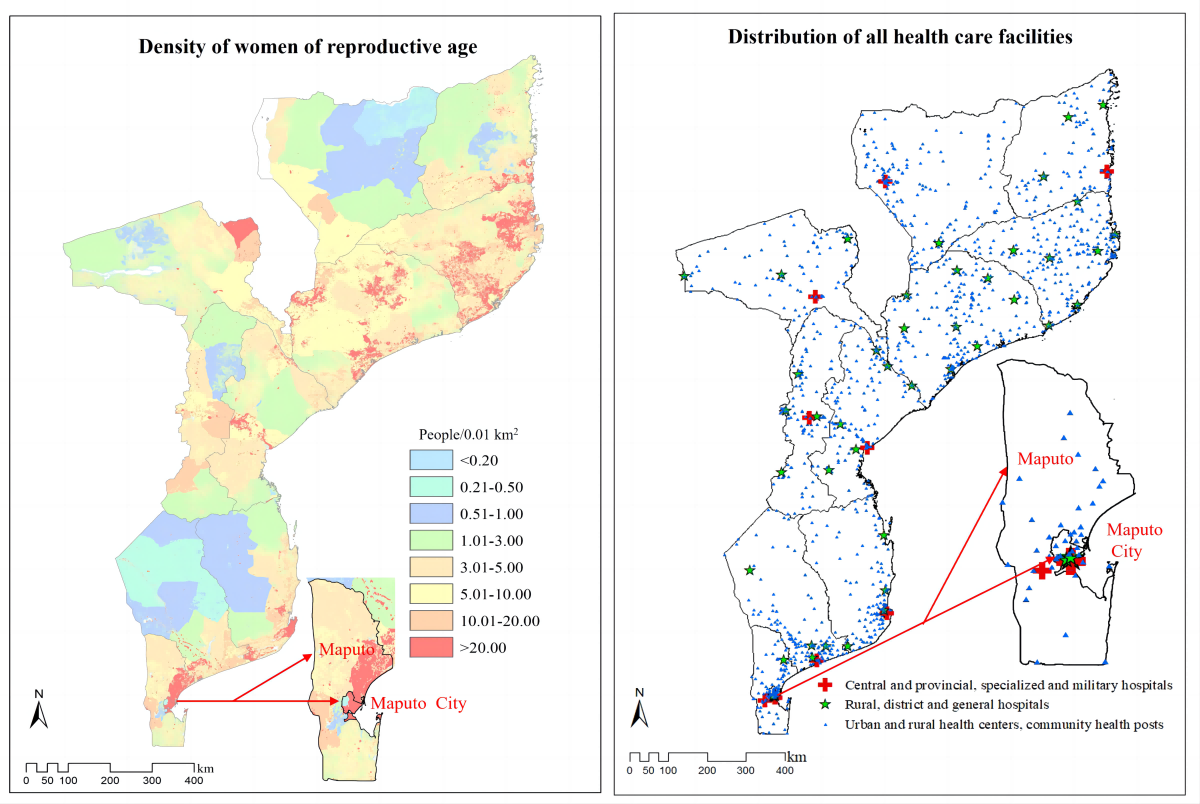
Capability of delivering the 3 service packages of continuous maternal and perinatal health care services was identified for every health care facility on the supply side. The WorldPop data set was used to provide spatial distribution of pregnant women at a 1×1 km^2^ resolution on the demand side. (A) The spatial distribution of women of reproductive age (people/0.01 km^2^). (B) The spatial distribution of the 3 levels of health care facilities in Mozambique in 2018. Maputo and Maputo City are displayed in the figures separately.

Figure 2 shows the spatial distribution of the 3 levels of health care facilities able to deliver the 3 service packages. The spatial distribution of different levels of health care facilities able to provide ANC, ID, and PNC, respectively, is shown in Multimedia Appendix 1, Figure S2.

### Spatial Access to The 3 Service Packages of CMPHS

In Mozambique, the median shortest travel times needed to access ANC, ID, and PNC were 2.38 (IQR 1.38-3.89) hours, 3.69 (IQR 1.87-5.28) hours, and 4.16 (IQR 2.48-6.67) hours, respectively. The shortest travel times required to reach the nearest health care facilities offering these services varied significantly across different regions. For ANC services, the shortest travel time ranged from 0.46 (IQR 0.26-0.69) hours in Maputo City to 4.95 (IQR 3.03-7.21) hours in Manica province. For ID services, it ranged from 0.74 (IQR 0.47-1.04) hours in Maputo City to 18.20 (IQR 11.67-24.65) hours in Niassa province. For PNC services, the shortest travel time ranged from 1.34 (IQR 0.83-1.85) hours in Maputo City to 10.76 (IQR 7.53-13.66) hours in Inhambane province. Except for Manica province, the shortest travel time needed to access ID was greater than for ANC in all other provinces. The shortest travel time spent to access ID was found to be distinctly longer than that for PNC in 3 provinces, namely Gaza, Maputo, and Niassa (Table 3).

**Figure 3:**
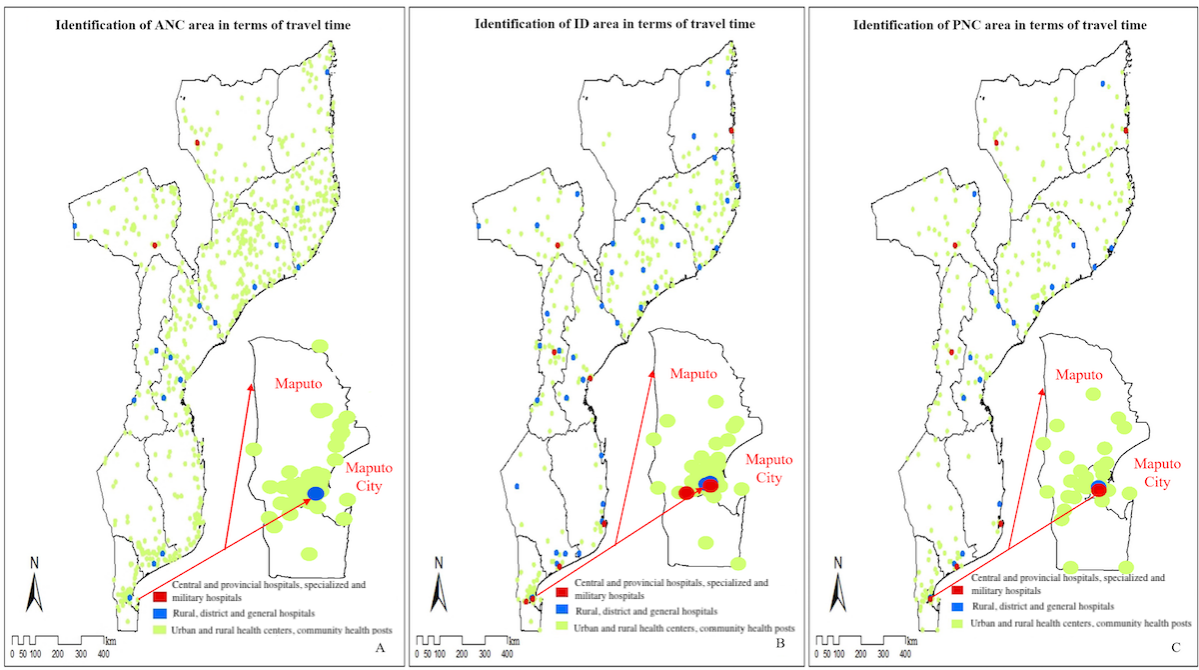
The spatial coverage of antenatal care (ANC), institutional delivery (ID), and postnatal care (PNC) provided by different levels of health care facilities by walking along a straight line with a speed of 4 km/hour and the standard of 2-hour security service range. The coverage area (in terms of 2-hour travel time) for (A) ANC, (B) ID, and (C) PNC. Maputo and Maputo City are displayed in the figures separately.

The results for coverage of different services in Figure 3 shows that women of reproductive age living in Maputo City were able to obtain timely ANC, ID, and PNC. Conversely, multiple provinces with lower population density were recognized as underserved areas, including the provinces of Niassa, Cabo Delgado, Gaza, and Inhambane, especially for the provision of ID and PNC.

**Table 3:** The shortest travel time spent to reach antenatal care, institutional delivery, and postnatal care was calculated for different regions using the nearest-neighbor method with a criterion of 2 hours’ walking, which was set under the scenario that demanders walked along a straight line with a speed of 4 km/hour.

Region	Time to reach antenatal care (hours)		Time to reach institutional delivery (hours)		Time to reach postnatal care (hours)	
	Median (IQR)	Maximum	Median (IQR)	Maximum	Median (IQR)	Maximum
						
Mozambique	2.38 (1.38-3.89)	30.38	3.69 (1.87-5.28)	60.27	4.16 (2.48-6.67)	45.53
Cabo Delgado	4.01 (2.39-6.82)	25.71	4.89 (2.89-8.40)	33.66	4.78 (2.64-8.11)	29.78
Gaza	2.90 (1.56-4.25)	20.40	6.09 (3.71-8.64)	27.62	4.39 (2.71-6.35)	27.99
Inhambane	2.69 (1.49-3.91)	16.95	4.43 (2.37-7.16)	31.46	10.76 (7.53-13.66)	33.82
Manica	4.95 (3.03-7.21)	27.48	3.17 (1.94-4.41)	20.05	4.09 (2.67-5.67)	20.05
Maputo	1.94 (1.03-3.02)	18.92	3.07 (1.78-4.79)	18.61	2.33 (1.37-3.53)	18.14
Maputo City	0.46 (0.26-0.69)	2.19	0.74 (0.47-1.04)	2.31	1.34 (0.83-1.85)	3.70
Nampula	2.13 (1.37-2.80)	10.61	2.81 (1.77-4.08)	13.81	4.37 (2.80-6.39)	17.15
Niassa	4.07 (2.41-6.63)	30.38	18.20 (11.67-24.65)	60.27	7.69 (4.74-13.05)	44.27
Sofala	2.11 (1.29-3.10)	18.16	4.90 (2.98-7.22)	25.21	4.21 (2.46-6.25)	22.28
Tete	3.87 (2.41-5.34)	18.06	5.70 (3.58-8.19)	23.86	5.51 (3.41-8.51)	45.53
Zambezia	2.47 (1.52-3.63)	12.83	3.78 (2.35-5.69)	19.66	4.03 (2.56-5.95)	16.76

### Classification and Identification of Underserved Areas for CMPHS

For women of reproductive age in Mozambique, more than 21% (living in about 2.69% of Mozambique’s area) lived in type 1 areas (able to obtain timely CMPHS including ANC, ID, and PNC); more than 51% (living in 83.25% of Mozambique’s area) lived in type 8 areas (not able to obtain timely access to any service packages of CMPHS); only 27.5% (living in about 14.07% of Mozambique’s area) lived in type 2-7 areas (able to access 1 or 2 types of services of CMPHS). The second highest percentage of women of reproductive age fell within type 5 areas, indicating that approximately 10% (living in about 7.42% of Mozambique’s area) were able to obtain ANC services in a timely manner. The third highest percentage of women of reproductive age fell within type 2 areas, meaning that more than 9% (living in about 2.19% of Mozambique’s area) were able to reach ANC and PNC in a timely manner (Table 4).

**Table 4:** Three layers representing timely access to antenatal care (ANC), institutional delivery (ID), and postnatal care (PNC), respectively, were overlapped to generate 8 types of multilevel health care access zones. The percentages by land area and by women of reproductive age covered within each type of multilevel health care access zone were calculated using an overlap analysis with a criterion of 2-hour security service range.

Type	Multilevel health care access zones	Land area, %	Population, %
1	Able to obtain timely CMPHS^a^, including ANC, ID, and PNC	2.69	21.1
2	Able to obtain timely ANC and ID	2.19	9.7
3	Able to obtain timely ANC and PNC	1.61	3.79
4	Able to obtain timely ID and PNC	0.95	1.63
5	Able to obtain timely ANC	7.42	9.96
6	Able to obtain timely ID	1.03	1.47
7	Able to obtain timely PNC	0.87	0.95
8	Not able to obtain timely CMPHS, including ANC, ID, and PNC	83.25	51.4

^a^CMPHS: continuous maternal and perinatal health care services.

**Figure 4:**
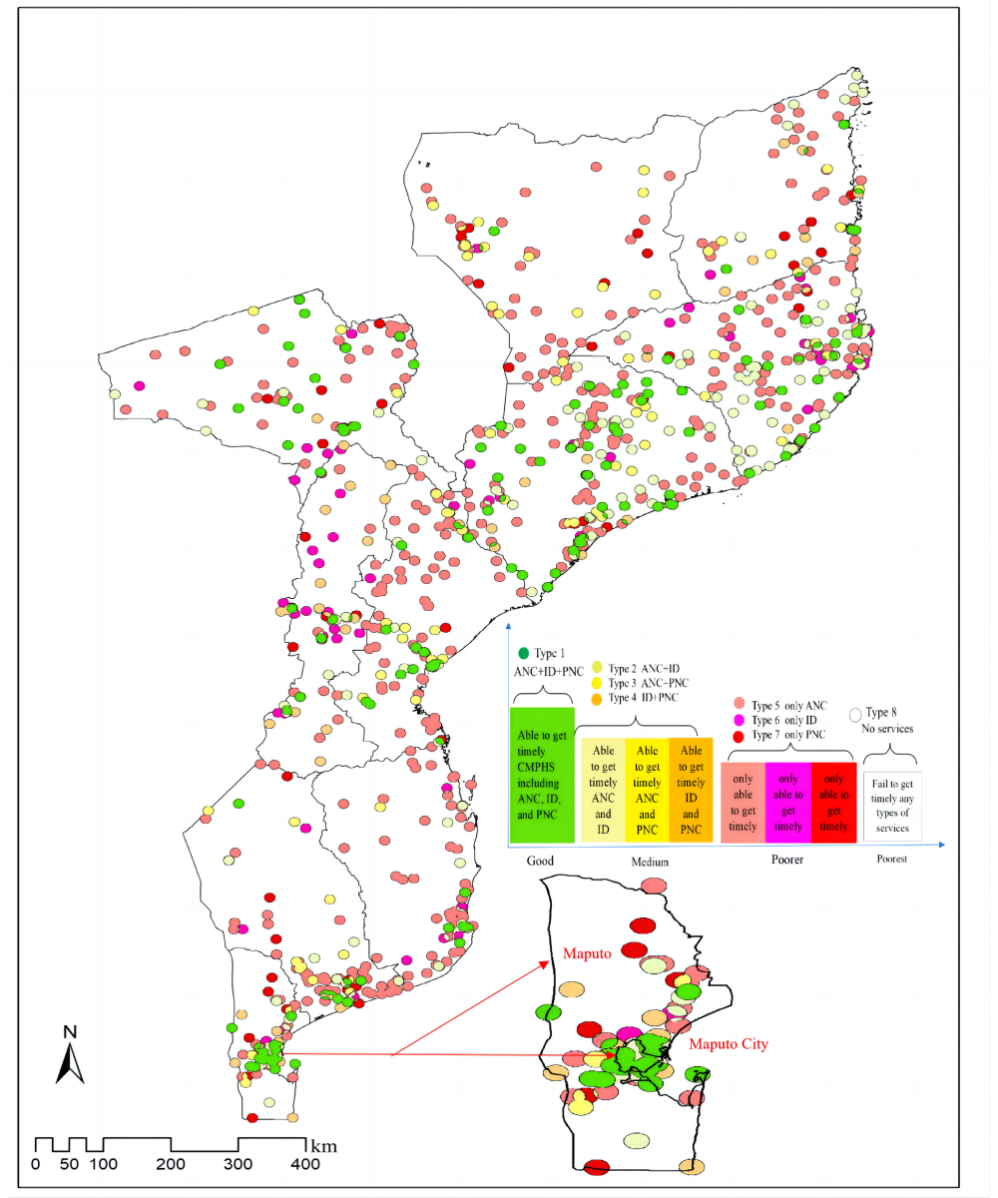
Overlay analysis of spatial access among women of reproductive age to continuous maternal and perinatal health care services (CMPHS) with a criterion of a 2-hour secure service range. Multilevel health care access zones are areas able to access all 3 service packages, 2 of the 3 service packages, only 1 of the 3 service packages, or none of the 3 service packages. Green is used to represent full access to CMPHS (ie, a good service type, namely type 1), shades of yellow are used to represent access to any 2 types of service (ie, medium service types, namely type 2, type 3, and type 4), shades of red are used to represent access to any 1 type of service (ie, poorer service types, namely type 5, type 6, and type 7), and the blank areas represent no access to any type of services (ie, the poorest service type, namely type 8). Maputo and Maputo City are displayed in the figure separately. ANC: antenatal care; ID: institutional delivery; PNC: postnatal care.

As can be seen in Figure 4 and Multimedia Appendix 1, Figure S3, the coverage of CMPHS was low in Mozambique, especially in the provinces of Inhambane, Niassa, and Gaza. Maputo City was the only region that was able to provide timely CMPHS for women of reproductive age.

## Discussion

### Principal Findings

Our study proposed a 2-step procedure to assess the spatial accessibility of CMPHS and verified its applicability through a case study in Mozambique. The results first showed spatial access to different types of CMPHS and disparities among different service packages. By using an overlay analysis, this procedure could identify 7 types of underserved areas and the detailed spatial distribution of existing well-served areas. The results from this procedure have evidence-based implications to inform decision-making processes related to health resource allocation, particularly in low-resource settings confronted with similar challenges. This can ultimately aid in improving maternal and newborn health outcomes. The necessity of enhancing spatial accessibility as a critical solution to improve coverage of CMPHS is also highlighted. In the specific case of Mozambique, only 21% of women of reproductive age could access CMPHS in a timely manner, and these women were predominately clustered in the most-developed region, Maputo City. This highlights that spatial access to CMPHS remains a significant challenge and that improvements in the coverage of ID and PNC are urgently needed.

Large disparities among regions in accessing CMPHS were also a critical issue. Inequities in CMPHS exist at the national, provincial, and district levels and always present fundamental barriers to progress, particularly among the most disadvantaged population groups [46]. This has been validated by our findings that the provinces of both Niassa and Cabo Delgado, which have lower population densities, tended to be more underserved, especially in accessing ID and PNC service packages. Our study also found that Maputo City was the only administrative unit that was capable of providing effective access to all the 3 service packages. We assume this is mainly because Maputo City is the capital of Maputo and the economic and social development center of Mozambique, so its development status far exceeds all the other areas [47]. In addition, the frequent occurrence of natural disasters, which greatly hinders health care service delivery, further adds to health inequities between the southern and northern regions in Mozambique, leading to lower rates of both ANC and ID visits in the southern region, corresponding to 74 additional maternal deaths and 726 additional deaths of children under the age of 1 month by the end of 2021 [48].

In the context of Mozambique’s poor accessibility and low equity in health care, the implementation of a decentralized allocation strategy to address the inequity issue has been recognized as an effective measure to promote both accessibility and coverage of CMPHS, which can take China’s successful experiences as an example [49,50]. China has made remarkable achievements and passed milestones in achieving SDGs through implementing a decentralized allocation strategy to improve accessibility and equality of health care services. China clearly indicated that women and children were target groups in the cooperation for global health in the Belt and Road Initiative in 2017 and implemented 100 foreign assistance programs for women and children’s health in low-income countries, so decentralized allocation strategies from China can help low-income countries improve their own CMPHS [51]. For example, strategies for the promotion of primary health care, along with the optimization of a hierarchical health care delivery system intended for maternal and child health care, were implemented. According to these strategies, integrated service packages delivered via the collaborative efforts of tertiary hospitals and primary maternal health care facilities proved to be an effective solution to strengthen the capacity of local health care facilities, as well as their corresponding referral systems, which resulted in increased in-hospital births and significantly reduced maternal mortality [50,52,53]. Furthermore, community-based interventions with components to engage or mobilize communities have been increasingly implemented to improve maternal and newborn health outcomes in low-resource settings [54-56]. For example, training programs should be provided to cultivate highly skilled health care professionals among local health care facilities [55,57].

### Comparison With Prior Work

Few studies have assessed the spatial accessibility of health care resources in Mozambique. A previous study reported that 67.3% of Mozambique’s population lived outside a 1-hour travel distance from primary health care centers [58]. However, spatial barriers to accessing health care services are gaining increasing attention. According to the *Statistical Yearbook 2018 of Mozambique*, the average travel distance needed to access a health care facility for people living in remote areas was 12.3 km [34]. The 2018-2019 Mozambique Humanitarian Response Plan reported that about 50% of the population lived more than 20 km away from the nearest health care facility [59]. Given the national focus on maternal and perinatal health, studies assessing spatial access to CMPHS have been carried out in the context of low-resource settings [14,17,60]. Previous findings suggest that 49.8% of women of reproductive age in Mozambique lived outside a 2-hour travel area from the nearest hospital for emergency services [61]. In southern Mozambique, 46% of pregnant women were able to access the nearest primary health care facility within 1 hour by walking, while 64% of women living in the region could receive life-saving service delivery within 2 hours [46]. While most existing literature has only assessed spatial access to single services, our results are consistent with previous findings that spatial access to maternal and perinatal health care services is poor in Mozambique. Moreover, our study further considered 3 separate service packages, representing important stages in the continuity of maternal and perinatal health care services, revealing spatial access to CMPHS in a straightforward manner.

### Public Health Implications

Our findings provide important evidence on the development of tailored, country-specific policies and interventions to address the geographical disparities of CMPHS in low-resource settings, which can support the development of resource allocation strategies based on the priority of CMPHS delivery. Using Mozambique as a case study of a low-resource setting, we recommend that public health policy makers and decision-makers draw from successful lessons such as decentralized allocation strategies. Through a comprehensive understanding of the geographical barriers to access to CMPHS, these strategies can quickly and efficiently improve maternal and neonatal health outcomes and contribute to the achievement of SDG3 and UHC.

### Limitations

This study does have several limitations. First, we set a scenario of women of reproductive age walking to health care facilities to reflect the most basic requirement of the vast number of vulnerable people in Mozambique. Other travel modes were not considered due to data limitations and the complexity of factors influencing the choice of travel mode. Specific travel thresholds for different levels of health care facilities were also not set due to a lack of reference data. Second, we used the spatial distribution of women of reproductive age as demand-side data instead of pregnancies due to data availability. Third, the integration of indicators representing the 3 service packages failed to take into account the type of health care facilities providing each service. Consequently, the continuum of services in different health care facilities was not further analyzed.

### Conclusions

Using Mozambique as a case study, this research used a 2-step procedure to assess the accessibility of CMPHS and proposes targeted policy suggestions for improving health outcomes. Further studies could be conducted, especially in low-resource settings, with reference to local context, and thus inform resource allocation–related decision-making procedures. In turn, this could potentially contribute to the improvement of global maternal and neonatal health, the realization of SDG3, and the attainment of UHC.

## Appendix

Multimedia Appendix 1 Supplementary Tables S1-S3 and Figures S1-S3.

## Abbreviations

ANC: antenatal care
CMPHS: continuous maternal and perinatal health care services
ID: institutional delivery
MNCH: maternal, newborn, and child health
NNM: nearest-neighbor method
PCPNC: pregnancy, childbirth, postpartum and newborn care
PNC: postnatal care
SARA: Service Availability and Readiness Assessment
SDG3: sustainable development goal 3
SSA: sub-Saharan Africa
UHC: universal health coverage
WHO: World Health Organization

## Data Availability

Data from the National Survey on Infrastructure, Equipment, Human Resources and Health Services 2018 can be accessed with approval from Mozambique’s Ministry of Health. Other data used in this study come from publicly available data sets with sources listed in the main text.
