# Supplementary material for: Spatial Access to Continuous Maternal and Perinatal Health Care Services in Low-Resource Settings: Cross-Sectional Study
Source: JMIR Public Health Surveill. 2024 Jul 18;10:e49367. doi: 10.2196/49367 (PMC11294765; doi:10.2196/49367)

# Spatial Access to Continuous Maternal and Perinatal Healthcare Services in Mozambique: A Cross-Sectional Study

## Appendix

## Methods

### Study Area

**Figure S1.** Administrative boundary of 11 provinces, population distribution (people/0.01km^2^), and average consumption levels (dollar/person/day) in Mozambique. (A) Administrative boundary. Data of administrative boundary was extracted from OpenStreetMap. (B) Population density. Data for population distribution was extracted from WorldPop 2015. (C) Average consumption levels. Data for average consumption levels was extracted from Mozambique’s Ministry of Economics and Finance in 2016.


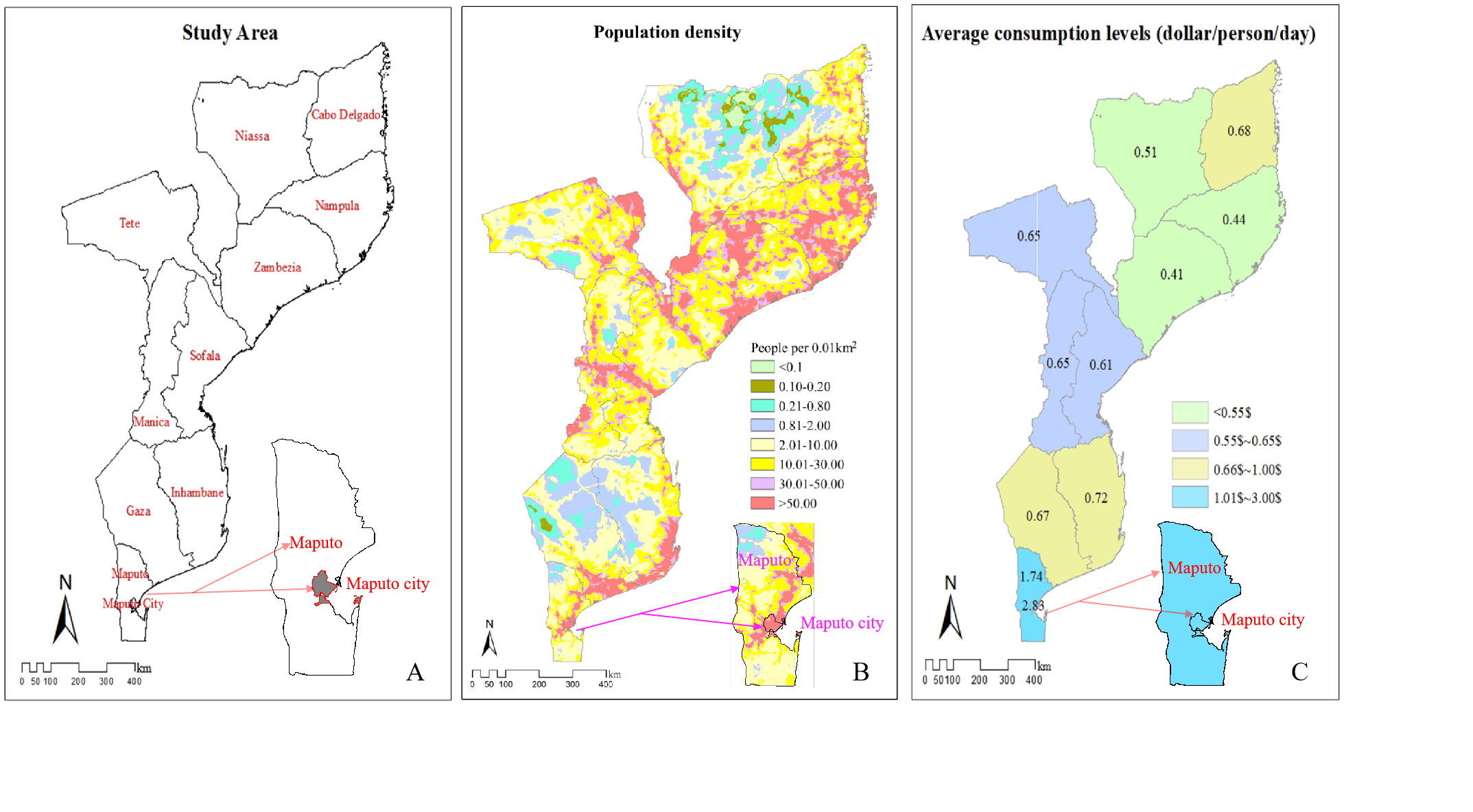


### Data pretreatment and analysis

This study aims to propose a procedure revealing spatial accessibility of CMPHS, which includes two steps that are indicators identification and spatial accessibility calculation, and two perspectives of data were needed, namely data for identifying indicators reflective of the delivery of three service packages in Mozambique, and data for spatial accessibility calculation.

#### Step One: Identification of indicators representing three service packages of CMPHS

Firstly, data extracted from the National Survey on Infrastructure, Equipment, Human Resources and Health Services 2018 which included a census of all healthcare facilities in the country and collected data through interviews with key informants at healthcare facilities using the core standard Service Availability and Readiness Assessment (SARA) questionnaire of WHO, but was adapted to the Mozambique local context for identification of indicators representing CMPHS in this study (Table S1).

**Table S1.** Number and percent of different levels of healthcare facilities capable to provide separate services (indicators)

| Indicators | Central and provincial hospitals, specialized and military hospitals (N=9, %) | Rural, district and general hospitals (N=43, %) | Urban and rural health centers, community health posts (N=1490, %) | Total (N=1542, %) | Selected indicators for  this study  (Yes or No) |
| --- | --- | --- | --- | --- | --- |
| **Antenatal care** |  |  |  |  |  |
| Iron supplementation |  |  |  |  | **Yes** |
| Yes | 2 (22.2) | 16 (37.2) | 1052 (70.6) | 1070 (69.4) |  |
| No | 7 (77.8) | 27 (62.8) | 438 (29.4) | 472 (30.6) |  |
| Folic acid supplementation |  |  |  |  | **Yes** |
| Yes | 2 (22.2) | 18 (41.9) | 1015 (68.1) | 1035 (67.1) |  |
| No | 7 (77.8) | 25 (58.1) | 475 (31.9) | 507 (32.9) |  |
| IPTp^a^ for malaria |  |  |  |  | **Yes** |
| Yes | 2 (22.2) | 19 (44.2) | 1387 (93.1) | 1408 (91.3) |  |
| No | 7 (77.8) | 24 (55.8) | 103 (6.9) | 134 (8.7) |  |
| Tetanus toxoid vaccination |  |  |  |  | **Yes** |
| Yes | 1 (11.1) | 19 (44.2) | 1344 (90.2) | 1364 (88.5) |  |
| No | 8 (88.9) | 24 (55.8) | 146 (9.8) | 178 (11.5) |  |
| Monitoring for hypertensive disorder of pregnancy |  |  |  |  | **Yes** |
| Yes | 2 (22.2) | 19 (44.2) | 1177 (79.0) | 1198 (77.7) |  |
| No | 7 (77.8) | 24 (55.8) | 313 (21.0) | 344 (22.3) |  |
| Guidelines ANC |  |  |  |  | No |
| Yes | 1 (11.1) | 13 (30.2) | 558 (37.4) | 572 (37.1) |  |
| No | 8 (88.9) | 30 (69.8) | 932 (62.6) | 970 (62.9) |  |
| ANC check-lists |  |  |  |  | No |
| Yes | 0 (0.0) | 13 (30.2) | 735 (49.3) | 748 (48.5) |  |
| No | 9 (100.0) | 30 (69.8) | 755 (50.7) | 794 (51.5) |  |
| Guidelines IPTp |  |  |  |  | No |
| Yes | 0 (0.0) | 13 (30.2) | 536 (36.0) | 549 (35.6) |  |
| No | 9 (100.0) | 30 (69.8) | 954 (64.0) | 993 (64.4) |  |
| Staff trained ANC |  |  |  |  | No |
| Yes | 1 (11.1) | 10 (23.3) | 548 (36.8) | 559 (36.3) |  |
| No | 8 (88.9) | 33 (76.7) | 942 (63.2) | 983 (63.7) |  |
| Staff trained IPTp |  |  |  |  | No |
| Yes | 0 (0.0) | 9 (20.9) | 463 (31.1) | 472 (30.6) |  |
| No | 9 (100.0) | 34 (79.1) | 1027 (68.9) | 1070 (69.4) |  |
| PMTCT^b^ services offered |  |  |  |  | No |
| Yes | 6 (66.7) | 29 (67.4) | 1340 (89.9) | 1375 (89.2) |  |
| No | 3 (33.3) | 14 (32.6) | 150 (10.1) | 167 (10.8) |  |
| HIV counseling and testing to HIV+ pregnant women |  |  |  |  | **Yes** |
| Yes | 6 (66.7) | 29 (67.4) | 1334 (89.5) | 1369 (88.8) |  |
| No | 3 (33.3) | 14 (32.6) | 156 (10.5) | 173 (11.2) |  |
| Antiviral treatment for HIV+ pregnant women |  |  |  |  | **Yes** |
| Yes | 5 (55.6) | 27 (62.8) | 1315 (88.3) | 1347 (87.4) |  |
| No | 4 (44.4) | 16 (37.2) | 175 (11.7) | 195 (12.6) |  |
| Nutritional counseling for HIV+ women and their infants |  |  |  |  | No |
| Yes | 5 (55.6) | 28 (65.1) | 1336 (89.7) | 1369 (88.8) |  |
| No | 4 (44.4) | 15 (34.9) | 154 (10.3) | 173 (11.2) |  |
| Family planning counseling to HIV+ pregnant women |  |  |  |  | No |
| Yes | 6 (66.7) | 28 (65.1) | 1332 (89.4) | 1366 (88.6) |  |
| No | 3 (33.3) | 15 (34.9) | 158 (10.6) | 176 (11.4) |  |
| Guidelines PMTCT |  |  |  |  | No |
| Yes | 4 (44.4) | 27 (62.8) | 1007 (67.6) | 1038 (67.3) |  |
| No | 5 (55.6) | 16 (37.2) | 483 (32.4) | 504 (32.7) |  |
| Staff trained PMTCT |  |  |  |  | No |
| Yes | 4 (44.4) | 21 (48.8) | 678 (45.5) | 703 (45.6) |  |
| No | 5 (55.6) | 22 (51.2) | 812 (54.5) | 839 (54.4) |  |
| Visual privacy for PMTCT |  |  |  |  | No |
| Yes | 0 (0.0) | 2 (4.7) | 116 (7.8) | 118 (7.7) |  |
| No | 9 (100.0) | 41 (95.3) | 1374 (92.2) | 1424 (92.3) |  |
| **Institutional delivery** |  |  |  |  |  |
| Administration of oxytocin injection |  |  |  |  | No |
| Yes | 8 (88.9) | 41 (95.3) | 1267 (85.0) | 1316 (85.3) |  |
| No | 1 (11.1) | 2 (4.7) | 223 (15.0) | 226 (14.7) |  |
| Monitoring of labour with partograph |  |  |  |  | **Yes** |
| Yes | 8 (88.9) | 41 (95.3) | 1224 (82.1) | 1273 (82.6) |  |
| No | 1 (11.1) | 2 (4.7) | 266 (17.9) | 269 (17.4) |  |
| Parenteral administration of antibiotics |  |  |  |  | No |
| Yes | 8 (88.9) | 41 (95.3) | 1037 (69.6) | 1086 (70.4) |  |
| No | 1 (11.1) | 2 (4.7) | 453 (30.4) | 456 (29.6) |  |
| Parenteral administration of oxytocic |  |  |  |  | **Yes** |
| Yes | 8 (88.9) | 40 (93.0) | 1195 (80.2) | 1243 (80.6) |  |
| No | 1 (11.1) | 3 (7.0) | 295 (19.8) | 299 (19.4) |  |
| Parenteral administration of magnesium sulphate |  |  |  |  | **Yes** |
| Yes | 8 (88.9) | 39 (90.7) | 1032 (69.3) | 1079 (70.0) |  |
| No | 1 (11.1) | 4 (9.3) | 458 (30.7) | 463 (30.0) |  |
| Assisted vaginal delivery |  |  |  |  | **Yes** |
| Yes | 8 (88.9) | 40 (93.0) | 1077 (72.3) | 1125 (73.0) |  |
| No | 1 (11.1) | 3 (7.0) | 413 (27.7) | 417 (27.0) |  |
| Emergency obstetric care |  |  |  |  | **Yes** |
| Yes | 8 (88.9) | 41 (95.3) | 455 (30.5) | 504 (32.7) |  |
| No | 1 (11.1) | 2 (4.7) | 1035 (69.5) | 1038 (67.3) |  |
| Manual removal of placenta |  |  |  |  | **Yes** |
| Yes | 8 (88.9) | 40 (93.0) | 1055 (70.8) | 1103 (71.5) |  |
| No | 1 (11.1) | 3 (7.0) | 435 (29.2) | 439 (28.5) |  |
| Removal of retained products after delivery |  |  |  |  | No |
| Yes | 8 (88.9) | 41 (95.3) | 1079 (72.4) | 1128 (73.2) |  |
| No | 1 (11.1) | 2 (4.7) | 411 (27.6) | 414 (26.8) |  |
| Cesarean section |  |  |  |  | No |
| Yes | 8 (88.9) | 36 (83.7) | 9 (0.6) | 53 (3.4) |  |
| No | 1 (11.1) | 7 (16.3) | 1481 (99.4) | 1489 (96.6) |  |
| Blood transfusion |  |  |  |  | No |
| Yes | 8 (88.9) | 39 (90.7) | 114 (7.7) | 161 (10.4) |  |
| No | 1 (11.1) | 4 (9.3) | 1376 (92.3) | 1381 (89.6) |  |
| Antibiotics for preterm |  |  |  |  | **Yes** |
| Yes | 8 (88.9) | 39 (90.7) | 802 (53.8) | 849 (55.1) |  |
| No | 1 (11.1) | 4 (9.3) | 688 (46.2) | 693 (44.9) |  |
| Corticosteroids in preterm labour |  |  |  |  | No |
| Yes | 7 (77.8) | 37 (86.0) | 283 (19.0) | 327 (21.2) |  |
| No | 2 (22.2) | 6 (14.0) | 1207 (81.0) | 1215 (78.8) |  |
| Guidelines essential childbirth care |  |  |  |  | No |
| Yes | 6 (66.7) | 37 (86.0) | 619 (41.5) | 662 (42.9) |  |
| No | 3 (33.3) | 6 (14.0) | 871 (58.5) | 880 (57.1) |  |
| Check-lists for essential childbirth care |  |  |  |  | No |
| Yes | 6 (66.7) | 35 (81.4) | 715 (48.0) | 756 (49.0) |  |
| No | 3 (33.3) | 8 (18.6) | 775 (52.0) | 786 (51.0) |  |
| Staff trained essential childbirth care |  |  |  |  | No |
| Yes | 6 (66.7) | 24 (55.8) | 433 (29.1) | 463 (30.0) |  |
| No | 3 (33.3) | 19 (44.2) | 1057 (70.9) | 1079 (70.0) |  |
| Examination light- available |  |  |  |  | No |
| Yes | 6 (66.7) | 37 (86.0) | 470 (31.5) | 513 (33.3) |  |
| No | 3 (33.3) | 6 (14.0) | 1020 (68.5) | 1029 (66.7) |  |
| Examination light- functioning |  |  |  |  | No |
| Yes | 6 (66.7) | 29 (67.4) | 374 (25.1) | 409 (26.5) |  |
| No | 3 (33.3) | 14 (32.6) | 1116 (74.9) | 1133 (73.5) |  |
| Delivery pack- available |  |  |  |  | No |
| Yes | 8 (88.9) | 41 (95.3) | 1189 (79.8) | 1238 (80.3) |  |
| No | 1 (11.1) | 2 (4.7) | 301 (20.2) | 304 (19.7) |  |
| Delivery pack- functioning |  |  |  |  | No |
| Yes | 8 (88.9) | 41 (95.3) | 1183 (79.4) | 1232 (79.9) |  |
| No | 1 (11.1) | 2 (4.7) | 307 (20.6) | 310 (20.1) |  |
| Cord clamp- available |  |  |  |  |  |
| Yes | 5 (55.6) | 34 (79.1) | 1069 (71.7) | 1108 (71.9) |  |
| No | 4 (44.4) | 9 (20.9) | 421 (28.3) | 434 (28.1) |  |
| Cord clamp-functioning |  |  |  |  | No |
| Yes | 5 (55.6) | 34 (79.1) | 1068 (71.7) | 1107 (71.8) |  |
| No | 4 (44.4) | 9 (20.9) | 422 (28.3) | 435 (28.2) |  |
| Episiotomy scissors- available |  |  |  |  | No |
| Yes | 5 (55.6) | 39 (90.7) | 718 (48.2) | 762 (49.4) |  |
| No | 4 (44.4) | 4 (9.3) | 772 (51.8) | 780 (50.6) |  |
| Episiotomy scissors- functioning |  |  |  |  | No |
| Yes | 5 (55.6) | 38 (88.4) | 707 (47.4) | 750 (48.6) |  |
| No | 4 (44.4) | 5 (11.6) | 783 (52.6) | 792 (51.4) |  |
| Scissors or blade to cut cord- available |  |  |  |  | No |
| Yes | 7 (77.8) | 40 (93.0) | 1157 (77.7) | 1204 (78.1) |  |
| No | 2 (22.2) | 3 (7.0) | 333 (22.3) | 338 (21.9) |  |
| Scissors or blade to cut cord- functioning |  |  |  |  | No |
| Yes | 7 (77.8) | 40 (93.0) | 1155 (77.5) | 1202 (78.0) |  |
| No | 2 (22.2) | 3 (7.0) | 335 (22.5) | 340 (22.0) |  |
| Suture material with needle- available |  |  |  |  | No |
| Yes | 8 (88.9) | 41 (95.3) | 1196 (80.3) | 1245 (80.7) |  |
| No | 1 (11.1) | 2 (4.7) | 294 (19.7) | 297 (19.3) |  |
| Needle holder- available |  |  |  |  | No |
| Yes | 7 (77.8) | 39 (90.7) | 1142 (76.6) | 1188 (77.0) |  |
| No | 2 (22.2) | 4 (9.3) | 348 (23.4) | 354 (23.0) |  |
| Needle holder- functioning |  |  |  |  | No |
| Yes | 7 (77.8) | 39 (90.7) | 1143 (76.7) | 1189 (77.1) |  |
| No | 2 (22.2) | 4 (9.3) | 347 (23.3) | 353 (22.9) |  |
| Manual vacuum extractor- available |  |  |  |  | No |
| Yes | 7 (77.8) | 28 (65.1) | 785 (52.7) | 820 (53.2) |  |
| No | 2 (22.2) | 15 (34.9) | 705 (47.3) | 722 (46.8) |  |
| Manual vacuum extractor- functioning |  |  |  |  | No |
| Yes | 6 (66.7) | 27 (62.8) | 723 (48.5) | 756 (49.0) |  |
| No | 3 (33.3) | 16 (37.2) | 767 (51.5) | 786 (51.0) |  |
| Vacuum aspirator or kit- available |  |  |  |  | No |
| Yes | 8 (88.9) | 32 (74.4) | 218 (14.6) | 258 (16.7) |  |
| No | 1 (11.1) | 11 (25.6) | 1272 (85.4) | 1284 (83.3) |  |
| Vacuum aspirator or kit- functioning |  |  |  |  | No |
| Yes | 8 (88.9) | 29 (67.4) | 183 (12.3) | 220 (14.3) |  |
| No | 1 (11.1) | 14 (32.6) | 1307 (87.7) | 1322 (85.7) |  |
| Incubator- available |  |  |  |  | No |
| Yes | 5 (55.6) | 20 (46.5) | 37 (2.5) | 62 (4.0) |  |
| No | 4 (44.4) | 23 (53.5) | 1453 (97.5) | 1480 (96.0) |  |
| Incubator- functioning |  |  |  |  | No |
| Yes | 5 (55.6) | 15 (34.9) | 34 (2.3) | 54 (3.5) |  |
| No | 4 (44.4) | 28 (65.1) | 1456 (97.7) | 1488 (96.5) |  |
| Disposable latex gloves |  |  |  |  | No |
| Yes | 8 (88.9) | 36 (83.7) | 1106 (74.2) | 1150 (74.6) |  |
| No | 1 (11.1) | 7 (16.3) | 384 (25.8) | 392 (25.4) |  |
| Blank partograph |  |  |  |  | **Yes** |
| Yes | 8 (88.9) | 41 (95.3) | 1155 (77.5) | 1204 (78.1) |  |
| No | 1 (11.1) | 2 (4.7) | 335 (22.5) | 338 (21.9) |  |
| Delivery bed- available |  |  |  |  | No |
| Yes | 6 (66.7) | 31 (72.1) | 944 (63.4) | 981 (63.6) |  |
| No | 3 (33.3) | 12 (27.9) | 546 (36.6) | 561 (36.4) |  |
| Delivery bed-functioning |  |  |  |  | No |
| Yes | 6 (66.7) | 31 (72.1) | 897 (60.2) | 934 (60.6) |  |
| No | 3 (33.3) | 12 (27.9) | 593 (39.8) | 608 (39.4) |  |
| Blood pressure apparatus- available |  |  |  |  | No |
| Yes | 8 (88.9) | 40 (93.0) | 864 (58.0) | 912 (59.1) |  |
| No | 1 (11.1) | 3 (7.0) | 626 (42.0) | 630 (40.9) |  |
| Blood pressure apparatus- functioning |  |  |  |  | No |
| Yes | 8 (88.9) | 37 (86.0) | 805 (54.0) | 850 (55.1) |  |
| No | 1 (11.1) | 6 (14.0) | 685 (46.0) | 692 (44.9) |  |
| Clean running water- available |  |  |  |  | No |
| Yes | 8 (88.9) | 40 (93.0) | 1007 (67.6) | 1055 (68.4) |  |
| No | 1 (11.1) | 3 (7.0) | 483 (32.4) | 487 (31.6) |  |
| Hand washing soap- available |  |  |  |  | No |
| Yes | 7 (77.8) | 38 (88.4) | 847 (56.8) | 892 (57.8) |  |
| No | 2 (22.2) | 5 (11.6) | 643 (43.2) | 650 (42.2) |  |
| Alcohol based hand rub- available |  |  |  |  | No |
| Yes | 7 (77.8) | 37 (86.0) | 861 (57.8) | 905 (58.7) |  |
| No | 2 (22.2) | 6 (14.0) | 629 (42.2) | 637 (41.3) |  |
| Stock obstetric care meds |  |  |  |  | No |
| Yes | 8 (88.9) | 40 (93.0) | 1174 (78.8) | 1222 (79.2) |  |
| No | 1 (11.1) | 3 (7.0) | 316 (21.2%) | 320 (20.8) |  |
| Oxytocin injection |  |  |  |  | No |
| Yes | 8 (88.9) | 40 (93.0) | 1140 (76.5) | 1188 (77.0) |  |
| No | 1 (11.1) | 3 (7.0) | 350 (23.5) | 354 (23.0) |  |
| Oxytocin stored in cold storage |  |  |  |  | No |
| Yes | 6 (66.7) | 21 (48.8) | 109 (7.3) | 136 (8.8) |  |
| No | 3 (33.3) | 22 (51.2) | 1381 (92.7) | 1406 (91.2) |  |
| Health worker for caesarean section 24hrs |  |  |  |  | No |
| Yes | 8 (88.9) | 36 (83.7) | 7 (0.5) | 51 (3.3) |  |
| No | 1 (11.1) | 7 (16.3) | 1483 (99.5) | 1491 (96.7) |  |
| Anaesthetist 24hrs |  |  |  |  | No |
| Yes | 7 (77.8) | 34 (79.1) | 6 (0.4) | 47 (3.0) |  |
| No | 2 (22.2) | 9 (20.9) | 1484 (99.6) | 1495 (97.0) |  |
| Guidelines EmOC^c^ |  |  |  |  |  |
| Yes | 7 (77.8) | 28 (65.1) | 7 (0.5) | 42 (2.7) |  |
| No | 2 (22.2) | 15 (34.9) | 1483 (99.5) | 1500 (97.3) |  |
| Staff trained EmOC |  |  |  |  | No |
| Yes | 6 (66.7) | 23 (53.5) | 7 (0.5) | 36 (2.3) |  |
| No | 3 (33.3) | 20 (46.5) | 1483 (99.5) | 1506 (97.7) |  |
| **Postnatal care** |  |  |  |  |  |
| Immediate and exclusive breastfeeding |  |  |  |  | **Yes** |
| Yes | 8 (88.9) | 41 (95.3) | 1264 (84.8) | 1313 (85.1) |  |
| No | 1 (11.1) | 2 (4.7) | 226 (15.2) | 229 (14.9) |  |
| Hygienic cord care |  |  |  |  | **Yes** |
| Yes | 8 (88.9) | 41 (95.3) | 1258 (84.4) | 1307 (84.8) |  |
| No | 1 (11.1) | 2 (4.7) | 232 (15.6) | 235 (15.2) |  |
| Thermal protection |  |  |  |  | **Yes** |
| Yes | 8 (88.9) | 41 (95.3) | 1244 (83.5) | 1293 (83.9) |  |
| No | 1 (11.1) | 2 (4.7) | 246 (16.5) | 249 (16.1) |  |
| Neonatal resuscitation |  |  |  |  | **Yes** |
| Yes | 8 (88.9) | 41 (95.3) | 1073 (72.0) | 1122 (72.8) |  |
| No | 1 (11.1) | 2 (4.7) | 417 (28.0) | 420 (27.2) |  |
| Kangaroo mother care |  |  |  |  | **Yes** |
| Yes | 8 (88.9) | 38 (88.4) | 1048 (70.3) | 1094 (70.9) |  |
| No | 1 (11.1) | 5 (11.6) | 442 (29.7) | 448 (29.1) |  |
| Injectable antibiotics  for neonatal sepsis |  |  |  |  | **Yes** |
| Yes | 7 (77.8) | 37 (86.0) | 722 (48.5) | 766 (49.7) |  |
| No | 2 (22.2) | 6 (14.0) | 768 (51.5) | 776 (50.3) |  |
| Guidelines essential newborn care |  |  |  |  | No |
| Yes | 7 (77.8) | 35 (81.4) | 613 (41.1) | 655 (42.5) |  |
| No | 2 (22.2) | 8 (18.6) | 877 (58.9) | 887 (57.5) |  |
| Resuscitation table (with heat source)- available |  |  |  |  | No |
| Yes | 6 (66.7) | 39 (90.7) | 309 (20.7) | 354 (23.0) |  |
| No | 3 (33.3) | 4 (9.3) | 1181 (79.3) | 1188 (77.0) |  |
| Resuscitation table (with heat source)- functioning |  |  |  |  | No |
| Yes | 6 (66.7) | 38 (88.4) | 294 (19.7) | 338 (21.9) |  |
| No | 3 (33.3) | 5 (11.6) | 1196 (80.3) | 1204 (78.1) |  |
| Newborn bag and mask size 1 for term babies- available |  |  |  |  | No |
| Yes | 8 (88.9) | 39 (90.7) | 894 (60.0) | 941 (61.0) |  |
| No | 1 (11.1) | 4 (9.3) | 596 (40.0) | 601 (39.0) |  |
| Newborn bag and mask size 1 for term babies- functioning |  |  |  |  | No |
| Yes | 8 (88.9) | 38 (88.4) | 882 (59.2) | 928 (60.2) |  |
| No | 1 (11.1) | 5 (11.6) | 608 (40.8) | 614 (39.8) |  |
| Newborn bag and mask size 0 for pre-term babies- available |  |  |  |  | No |
| Yes | 8 (88.9) | 34 (79.1) | 598 (40.1) | 640 (41.5) |  |
| No | 1 (11.1) | 9 (20.9) | 892 (59.9) | 902 (58.5) |  |
| Newborn bag and mask size 0 for pre-term babies- functioning |  |  |  |  | No |
| Yes | 8 (88.9) | 34 (79.1) | 594 (39.9) | 636 (41.2) |  |
| No | 1 (11.1) | 9 (20.9) | 896 (60.1) | 906 (58.8) |  |
| Electric suction pump- available |  |  |  |  | No |
| Yes | 5 (55.6) | 14 (32.6) | 43 (2.9) | 62 (4.0) |  |
| No | 4 (44.4) | 29 (67.4) | 1447 (97.1) | 1480 (96.0) |  |
| Electric suction pump- functioning |  |  |  |  | No |
| Yes | 5 (55.6) | 13 (30.2) | 41 (2.8) | 59 (3.8) |  |
| No | 4 (44.4) | 30 (69.8) | 1449 (97.2) | 1483 (96.2) |  |
| Suction catheter- available |  |  |  |  | No |
| Yes | 5 (55.6) | 14 (32.6) | 163 (10.9) | 182 (11.8) |  |
| No | 4 (44.4) | 29 (67.4) | 1327 (89.1) | 1360 (88.2) |  |
| Suction catheter- functioning |  |  |  |  | No |
| Yes | 5 (55.6) | 15 (34.9) | 164 (11.0) | 184 (11.9) |  |
| No | 4 (44.4) | 28 (65.1) | 1326 (89.0) | 1358 (88.1) |  |
| Suction bulb, single use- available |  |  |  |  | No |
| Yes | 3 (33.3) | 9 (20.9) | 196 (13.2) | 208 (13.5) |  |
| No | 6 (66.7) | 34 (79.1) | 1294 (86.8) | 1334 (86.5) |  |
| Suction bulb, single use- functioning |  |  |  |  | No |
| Yes | 3 (33.3) | 8 (18.6) | 191 (12.8) | 202 (13.1) |  |
| No | 6 (66.7) | 35 (81.4) | 1299 (87.2) | 1340 (86.9) |  |
| Suction bulb, sterilizable multi-use- available |  |  |  |  | No |
| Yes | 7 (77.8) | 38 (88.4) | 1100 (73.8) | 1145 (74.3) |  |
| No | 2 (22.2) | 5 (11.6) | 390 (26.2) | 397 (25.7) |  |
| Suction bulb, sterilizable multi-use- functioning |  |  |  |  | No |
| Yes | 7 (77.8) | 38 (88.4) | 1086 (72.9) | 1131 (73.3) |  |
| No | 2 (22.2) | 5 (11.6) | 404 (27.1) | 411 (26.7) |  |
| Speculum- available |  |  |  |  | No |
| Yes | 8 (88.9) | 41 (95.3) | 1223 (82.1) | 1272 (82.5) |  |
| No | 1 (11.1) | 2 (4.7) | 267 (17.9) | 270 (17.5) |  |
| Speculum- functioning |  |  |  |  | No |
| Yes | 8 (88.9) | 41 (95.3) | 1224 (82.1) | 1273 (82.6) |  |
| No | 1 (11.1) | 2 (4.7) | 266 (17.9) | 269 (17.4) |  |
| Infant weighing scale- available |  |  |  |  | No |
| Yes | 8 (88.9) | 41 (95.3) | 1222 (82.0) | 1271 (82.4) |  |
| No | 1 (11.1) | 2 (4.7) | 268 (18.0) | 271 (17.6) |  |
| Infant weighing scale- functioning |  |  |  |  | No |
| Yes | 8 (88.9) | 41 (95.3) | 1196 (80.3) | 1245 (80.7) |  |
| No | 1 (11.1) | 2 (4.7) | 293 (19.6) | 296 (19.2) |  |
| Missing | 0 (0.0) | 0 (0.0) | 1 (0.1) | 1 (0.1) |  |
| Antibiotic eye ointment for newborn |  |  |  |  | No |
| Yes | 7 (77.8) | 39 (90.7) | 1005 (67.4) | 1051 (68.2) |  |
| No | 2 (22.2) | 4 (9.3) | 485 (32.6) | 491 (31.8) |  |
| Gentamicin injection |  |  |  |  | No |
| Yes | 8 (88.9) | 38 (88.4) | 648 (43.5) | 694 (45.0) |  |
| No | 1 (11.1) | 5 (11.6) | 842 (56.5) | 848 (55.0) |  |
| Ampicillin powder for injection |  |  |  |  | No |
| Yes | 8 (88.9) | 38 (88.4) | 634 (42.6) | 680 (44.1) |  |
| No | 1 (11.1) | 5 (11.6) | 856 (57.4) | 862 (55.9) |  |
| Hydralazine injection |  |  |  |  | No |
| Yes | 5 (55.6) | 36 (83.7) | 432 (29.0) | 473 (30.7) |  |
| No | 4 (44.4) | 7 (16.3) | 1058 (71.0) | 1069 (69.3) |  |
| Metronidazole injection |  |  |  |  | No |
| Yes | 8 (88.9) | 34 (79.1) | 586 (39.3) | 628 (40.7) |  |
| No | 1 (11.1) | 9 (20.9) | 904 (60.7) | 914 (59.3) |  |
| Azithromycin cap |  |  |  |  | No |
| Yes | 6 (66.7) | 28 (65.1) | 638 (42.8) | 672 (43.6) |  |
| No | 3 (33.3) | 15 (34.9) | 852 (57.2) | 870 (56.4) |  |
| Cefixime cap |  |  |  |  | No |
| Yes | 2 (22.2) | 17 (39.5) | 160 (10.7) | 179 (11.6) |  |
| No | 7 (77.8) | 26 (60.5) | 1330 (89.3) | 1363 (88.4) |  |
| Benzathine benzylpenicillin powder for injection |  |  |  |  | No |
| Yes | 4 (44.4) | 26 (60.5) | 588 (39.5) | 618 (40.1) |  |
| No | 5 (55.6) | 17 (39.5) | 902 (60.5) | 924 (59.9) |  |
| Nifedipine cap |  |  |  |  | No |
| Yes | 7 (77.8) | 38 (88.4) | 511 (34.3) | 556 (36.1) |  |
| No | 2 (22.2) | 5 (11.6) | 979 (65.7) | 986 (63.9) |  |
| Methyldopa tablet |  |  |  |  | No |
| Yes | 7 (77.8) | 36 (83.7) | 514 (34.5) | 557 (36.1) |  |
| No | 2 (22.2) | 7 (16.3) | 976 (65.5) | 985 (63.9) |  |
| Calcium gluconate injection |  |  |  |  | No |
| Yes | 4 (44.4) | 9 (20.9) | 57 (3.8) | 70 (4.5) |  |
| No | 5 (55.6) | 34 (79.1) | 1433 (96.2) | 1472 (95.5) |  |
| Magnesium sulphate injectable |  |  |  |  | No |
| Yes | 8 (88.9) | 37 (86.0) | 816 (54.8) | 861 (55.8) |  |
| No | 1 (11.1) | 6 (14.0) | 674 (45.2) | 681 (44.2) |  |
| Skin disinfectant |  |  |  |  | No |
| Yes | 8 (88.9) | 37 (86.0) | 984 (66.0) | 1029 (66.7) |  |
| No | 1 (11.1) | 6 (14.0) | 506 (34.0) | 513 (33.3) |  |
| Intravenous solution with infusion set |  |  |  |  | No |
| Yes | 7 (77.8) | 32 (74.4) | 732 (49.1) | 771 (50.0) |  |
| No | 2 (22.2) | 11 (25.6) | 758 (50.9) | 771 (50.0) |  |
| Sodium chloride injectable solution |  |  |  |  | No |
| Yes | 0 (0.0) | 0 (0.0) | 0 (0.0) | 0 (0.0) |  |
| No | 0 (0.0) | 0 (0.0) | 0 (0.0) | 0 (0.0) |  |
| Missing | 9 (100.0) | 43 (100.0) | 1490 (100.0) | 1542 (100.0) |  |
| Dexamethasone injection |  |  |  |  | No |
| Yes | 7 (77.8) | 31 (72.1) | 124 (8.3) | 162 (10.5) |  |
| No | 2 (22.2) | 12 (27.9) | 1366 (91.7) | 1380 (89.5) |  |
| IVG^d^ |  |  |  |  | No |
| Yes | 7 (77.8) | 27 (62.8) | 5 (0.3) | 39 (2.5) |  |
| No | 2 (22.2) | 16 (37.2) | 1485 (99.7) | 1503 (97.5) |  |
| Facility provide immunization  services today |  |  |  |  | No |
| Yes | 3 (33.3) | 16 (37.2) | 1278 (85.8) | 1297 (84.1) |  |
| No | 6 (66.7) | 27 (62.8) | 212 (14.2) | 245 (15.9) |  |
| Birth doses |  |  |  |  | No |
| Yes | 1 (11.1) | 14 (32.6) | 978 (65.6) | 993 (64.4) |  |
| No | 8 (88.9) | 29 (67.4) | 512 (34.4) | 549 (35.6) |  |
| Infant vaccines |  |  |  |  | No |
| Yes | 0 (0.0) | 15 (34.9) | 1020 (68.5) | 1035 (67.1) |  |
| No | 9 (100.0) | 28 (65.1) | 470 (31.5) | 507 (32.9) |  |
| Measles vaccine and diluent |  |  |  |  | No |
| Yes | 3 (33.3) | 9 (20.9) | 734 (49.3) | 746 (48.4) |  |
| No | 6 (66.7) | 32 (74.4) | 572 (38.4) | 610 (39.6) |  |
| Missing | 0 (0.0) | 2 (4.7) | 184 (12.3) | 186 (12.1) |  |
| DPT-Hib-HepB^e^ |  |  |  |  | No |
| Yes | 3 (33.3) | 11 (25.6) | 662 (44.4) | 676 (43.8) |  |
| No | 6 (66.7) | 31 (72.1) | 616 (41.3) | 653 (42.3) |  |
| Missing | 0 (0.0) | 1 (2.3) | 212 (14.2) | 213 (13.8) |  |
| Oral polio vaccine |  |  |  |  | No |
| Yes | 2 (22.2) | 9 (20.9) | 530 (35.6) | 541 (35.1) |  |
| No | 6 (66.7) | 30 (69.8) | 614 (41.2) | 650 (42.2) |  |
| Missing | 1 (11.1) | 4 (9.3) | 346 (23.2) | 351 (22.8) |  |
| BCG^f^ vaccine |  |  |  |  | Yes |
| Yes | 3 (33.3) | 11 (25.6) | 872 (58.5) | 886 (57.5) |  |
| No | 6 (66.7) | 32 (74.4) | 618 (41.5) | 656 (42.5) |  |
| Rotavirus vaccine |  |  |  |  | No |
| Yes | 2 (22.2) | 9 (20.9) | 537 (36.0) | 548 (35.5) |  |
| No | 7 (77.8) | 32 (74.4) | 633 (42.5) | 672 (43.6) |  |
| Missing | 0 (0.0) | 2 (4.7) | 320 (21.5) | 322 (20.9) |  |
| Pneumococcal vaccine |  |  |  |  | No |
| Yes | 1 (11.1) | 8 (18.6) | 424 (28.5) | 433 (28.1) |  |
| No | 7 (77.8) | 28 (65.1) | 635 (42.6) | 670 (43.5) |  |
| Missing | 1 (11.1) | 7 (16.3) | 431 (28.9) | 439 (28.5) |  |
| Inactivated polio vaccine |  |  |  |  | No |
| Yes | 2 (22.2) | 13 (30.2) | 755 (50.7) | 770 (49.9) |  |
| No | 7 (77.8) | 30 (69.8) | 735 (49.3) | 772 (50.1) |  |
| HIV counseling and testing to infants born to HIV+ women |  |  |  |  | **Yes** |
| Yes | 4 (44.4) | 26 (60.5) | 1321 (88.7) | 1351 (87.6) |  |
| No | 5 (55.6) | 17 (39.5) | 169 (11.3) | 191 (12.4) |  |
| ARV^g^ prophylaxis to newborns of HIV+ pregnant women |  |  |  |  | **Yes** |
| Yes | 6 (66.7) | 28 (65.1) | 1311 (88.0) | 1345 (87.2) |  |
| No | 3 (33.3) | 15 (34.9) | 179 (12.0) | 197 (12.8) |  |
| HIV+ infant and young child feeding counseling |  |  |  |  | **Yes** |
| Yes | 6 (66.7) | 28 (65.1) | 1333 (89.5) | 1367 (88.7) |  |
| No | 3 (33.3) | 15 (34.9) | 157 (10.5) | 175 (11.3) |  |
| Guidelines infant and young child feeding |  |  |  |  | No |
| Yes | 5 (55.6) | 22 (51.2) | 648 (43.5) | 675 (43.8) |  |
| No | 4 (44.4) | 21 (48.8) | 842 (56.5) | 867 (56.2) |  |
| Staff trained in infant and young child feeding |  |  |  |  | No |
| Yes | 6 (66.7) | 17 (39.5) | 510 (34.2) | 533 (34.6) |  |
| No | 3 (33.3) | 26 (60.5) | 980 (65.8) | 1009 (65.4) |  |

^a^IPTp: intermittent preventive treatment in pregnancy ^b^PMTCT: prevention of mother to child transmission

^c^EmOC: extracorporeal membrane oxygenation ^d^IVG: intravenous immunoglobulin

^e^DPT-Hib-HepB: Diphtheria- Haemophilus influenzae b-Hepatitis B (HepB)

^f^BCG: Bacillus Calmette-Guérin ^g^ARV: AIDS related virus

Secondly, adhere to the two main principles, recommended and available indicators were first filtered to identify a group of separate indicators from each single service package. However, it should be noted that in practice, it is difficult for indicators to be scaled up one by one due to the fact that in addition to limited essential indicators that are necessary and applicable everywhere, other indicators might be situational in endemic regions, and there are also indicators which have a small marginal effect to produce enough cost-effectiveness. With consideration of both avoiding fragmented services and ensuring the quality of CMPHS, as well as extensive reference to existing literatures, the filtered indicators were further screened and integrated to represent the three service packages covering the continuous periods for WoRA (Table S2).

**Table** **S2.** List of the finally included seven indicators for ANC, eight indicators for ID, ten indicators for PNC which represented the three service packages of CMPHS, and the corresponding selection process starting with an iniatial screening of indicators based on the guidelines proposed by WHO, then combing with data availability in Mozambique, and finalizing identifying of the indicator representing the three service packages with wide reference with existing literature.

| Three service packages | The global guidelines of MNCH^a^ and PCPNC^b^  proposed by WHO | Existing literatures | Selected indicators in Mozambique |
| --- | --- | --- | --- |
| Antenatal care (ANC) | Folic acid supplementation;  Iron supplementation;  IPTp^c^ and ITN^d^ for malaria;  Tetanus toxoid vaccination;  Measure blood pressure;  Pre-eclampsia and eclampsia prevention;  Prevention and management of STIs^e^ including HIV  PMTCT^f^ services including anti–retroviral therapy for pregnant women;  Detection and treatment of bacteriuria;  Detection and management of fetal growth restriction; Detection and management of diabetes in pregnancy; Counselling and preparation for newborn care and breastfeeding;  Prevention and management of TB^g^;  Prevention and management of maternal anaemia; | Folic acid supplementation;  Iron supplementation;  IPTp^c^ and ITN^d^ for malaria;  Tetanus injection;  Blood pressure measured;  Prevention and management of infectious diseases (malaria, HIV, tuberculosis);  Antenatal visits;  Weight and height measured;  Prevention and management of maternal anaemia;  Informed about pregnancy complication; Given malaria prophylaxi; | Folic acid supplementation;  Iron supplementation;  IPTp^c^ for malaria;  Tetanus toxoid vaccination;  Monitoring for hypertensive disorder of pregnancy;  HIV counseling and testing to HIV+ pregnant women;  Antiviral treatment to HIV+ pregnant women; |
| Institutional delivery (ID) | Skilled obstetric at birth/Skilled birth attendant;  Clean birth practices;  Birth and emergency preparedness;  Monitoring of labour with partograph;  Magnesium sulfate for eclampsia;  Antibiotics for preterm rupture of membranes; Corticosteroids for preterm labour;  Antenatal corticosteroids for preterm labor;  Caesarean section and prophylactic antibiotics;  Emergency obstetric care to manage complications; | Using skilled and institutional birth-care services;  Emergency obstetric care;  Training for traditional birth attendants on safe deliveries;  Delivery at the healthcare facility;  Utilization of caesarean section services; | Monitoring of labour with partograph; Parenteral administration of oxytocic; Assisted vaginal delivery;  Manual removal of placenta;  Antibiotics for preterm rupture of membranes;  Blank partograph;  Parenteral administration of magnesium sulphate;  Emergency obstetric care; |
| Postnatal care (PNC) | Immediate initiation of exclusive breastfeeding;  Immediate thermal care;  Resuscitation of newborn baby;  Emergency newborn care for sepsis;  LBW^h^ babies given kangaroo mother care;  Hygienic cord care and skin care;  Detect and manage sepsis;  Screen/initiate/continue ARV^i^ for HIV;  Case management of infections;  Case management for pneumonia;  Delay in bathing;  Immunization services; | Breastfeeding within 1h of birth newborn;  Thermal protection;  Neonatal resuscitation;  LBW babies given kangaroo mother care; BCG^j^ and polio vaccination;  Care of children with HIV;  Early detection and referral of complications;  PMTCT^f^ services including appropriate feeding;  Immediate emergency care for newborn babies; | Immediate and exclusive breastfeeding;  Thermal protection;  Neonatal resuscitation;  Staff trained newborn resuscitation; Kangaroo mother care;  Injectable antibiotics for neonatal sepsis; HIV counseling and testing to infants born to HIV+ women;  ARV^i^ prophylaxis to newborns of HIV+ pregnant women;  HIV+ infant and young child feeding counseling;  BCG^j^ vaccination; |

^a^MNCH: Maternal, Newborn, and Child Health (MNCH). ^b^PCPNC: Pregnancy, Childbirth, Postpartum and Newborn Care. ^c^IPTp: intermittent preventive treatment in pregnancy. ^d^ITN:insecticide–treated bednet. ^e^STIs: sexually transmitted diseases.  ^f^PMTCT: prevention of mother to child transmission. ^g^TB: tuberculosis. ^h^LBW: low-birth-weight. ^i^ARV: AIDS related virus. ^j^BCG: bacillus calmette-guérin.

#### Step Two: Calculation of spatial access to CMPHS

**Detailed description of demand side data.** grided spatial distribution of WoRA which is 15-49 women was utilized. With pregnant women being the most important and focused target population of CMPHS, the worldwide publicly available dataset WorldPop was utilized to provide spatial distribution of pregnant women at 1*1km^2^ resolution as the weight of spatial distribution of demand side data. Because the WorldPop announced data was estimated with national totals adjusted to match national estimates on pregnancies made by the Guttmacher but has not adjusted at lower administration level, and was only available for the year 2015, ideally, government announced pregnant women size in 2018 in Mozambique should be utilized to adjust the spatial distribution of pregnant women at 1*1km^2^ resolution to generate a more accurate and up-to-date demand distribution as generally processed in relevant research. However, publicly available data source for Mozambique only announced the number of population for different sex and age in 2018. Assuming that the possibility of WoRA got pregnant was nationwide uniform, the spatial distribution of WoRA should be the same with pregnant women. Meanwhile, taking WoRA as demand distribution, we also included family planning demand such as abortion which should also be provided by healthcare facilities as part of CMPHS. In conclusion, considering both data availability and research significance, we finally used the spatial distribution of WoRA as demand data. And it was generated by using the spatial distribution of pregnant women at 1*1km^2^ resolution from WorldPop as weight and downscaled the national announced provincial level aggregated number of 15-49 women with assumption that the fertility rate was uniform nationwide.

### Analysis of spatial accessibility

The travel mode of walking was utilized because according to data announced by Statistical Yearbook 2018 of Mozambique (Table S3), the total number of vehicles registered in Mozambique was only 47,693 and walking turned out to be the most available and possible travel mode for WoRA

**Table S3.** Vehicle registration data in Mozambique in 2018

| Regions | Number of registered vehicles | | | | | | Number of WoRA^a^ | Number of vehicles per thousand WoRA^a^ |
| --- | --- | --- | --- | --- | --- | --- | --- | --- |
|  | Total | Light vehicles | Heavy vehicles | Tractors | Trailers | Motorbike |  |  |
| Mozambique | 47693 | 35492 | 8489 | 260 | 1944 | 1508 | 6,318,836 | 7.548 |
| Niassa | 159 | 134 | 22 | - | 2 | 1 | 404,538 | 0.393 |
| Cabo Delgado | 990 | 461 | 513 | 0 | 15 | 1 | 528,400 | 1.874 |
| Nampula | 1059 | 683 | 312 | 6 | 26 | 32 | 1,306,024 | 0.811 |
| Zambezia | 91 | 49 | 35 | - | 2 | 5 | 1,190,510 | 0.076 |
| Tete | 535 | 243 | 212 | 1 | 58 | 21 | 605,529 | 0.884 |
| Manica | 214 | 89 | 74 | 13 | 22 | 16 | 455,704 | 0.470 |
| Sofala | 2032 | 343 | 691 | 71 | 384 | 543 | 538,717 | 3.772 |
| Inhambane | 733 | 591 | 94 | 2 | 29 | 17 | 364,153 | 2.013 |
| Gaza | 780 | 484 | 266 | 3 | - | 27 | 350,200 | 2.227 |
| Maputo | 11993 | 9554 | 1663 | 75 | 701 | 1159 | 32,279 | 371.542 |
| Maputo city | 29107 | 22861 | 4607 | 89 | 705 | 845 | 542,782 | 53.626 |

^a^WoRA: women of reproductive age. Data source: Statistical Yearbook 2018 of Mozambique

## Results

Figure S2. Spatial distribution of the three levels of healthcare facilities in Mozambique, which are urban and rural health centers, community health posts (the lowest level of healthcare facility), rural, district and general hositals (the middle level of healthcare facility), and central and provincial, specialized and military hospitals (the highest level of healthcare facility), capable to deliver ANC, ID, and PNC services. (A) Distribution of healthcare facilities delivering ANC (Antenatal care), (B) Distribution of healthcare facilities delivering ID (Institutional Delivery), and (C) Distribution of healthcare facilities delivering PNC (Postnatal care).


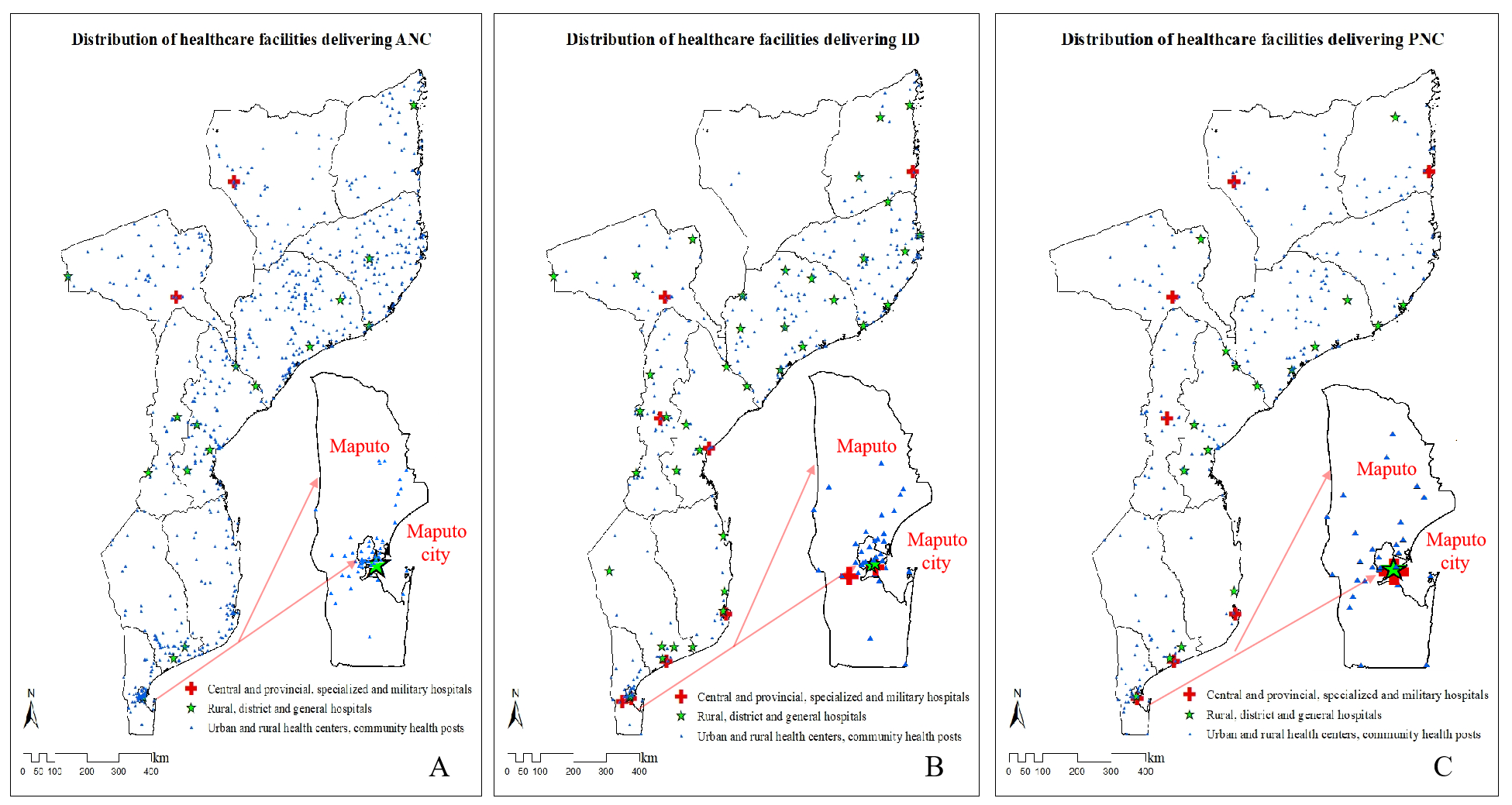


Figure S3. Spatial access to CMPHS in Mozambique was shown in Figure S3. Three layers representing timely access to ANC, ID, and PNC, respectively, were overlapped to generate 8 types of multi-level healthcare access zones. (A) Areas capable to access all three service packages, (B) Areas capable to access two out of the three service packages, and (C) Areas capable to access only one of the three service packages. Overall, as can be seen in Figure S3, WoRA living in Maputo city could get timely CMPHS including ANC, ID, and PNC. Conversely, under-served WoRA were mostly located in the provinces of Inhambane, Niassa, and Gaza.


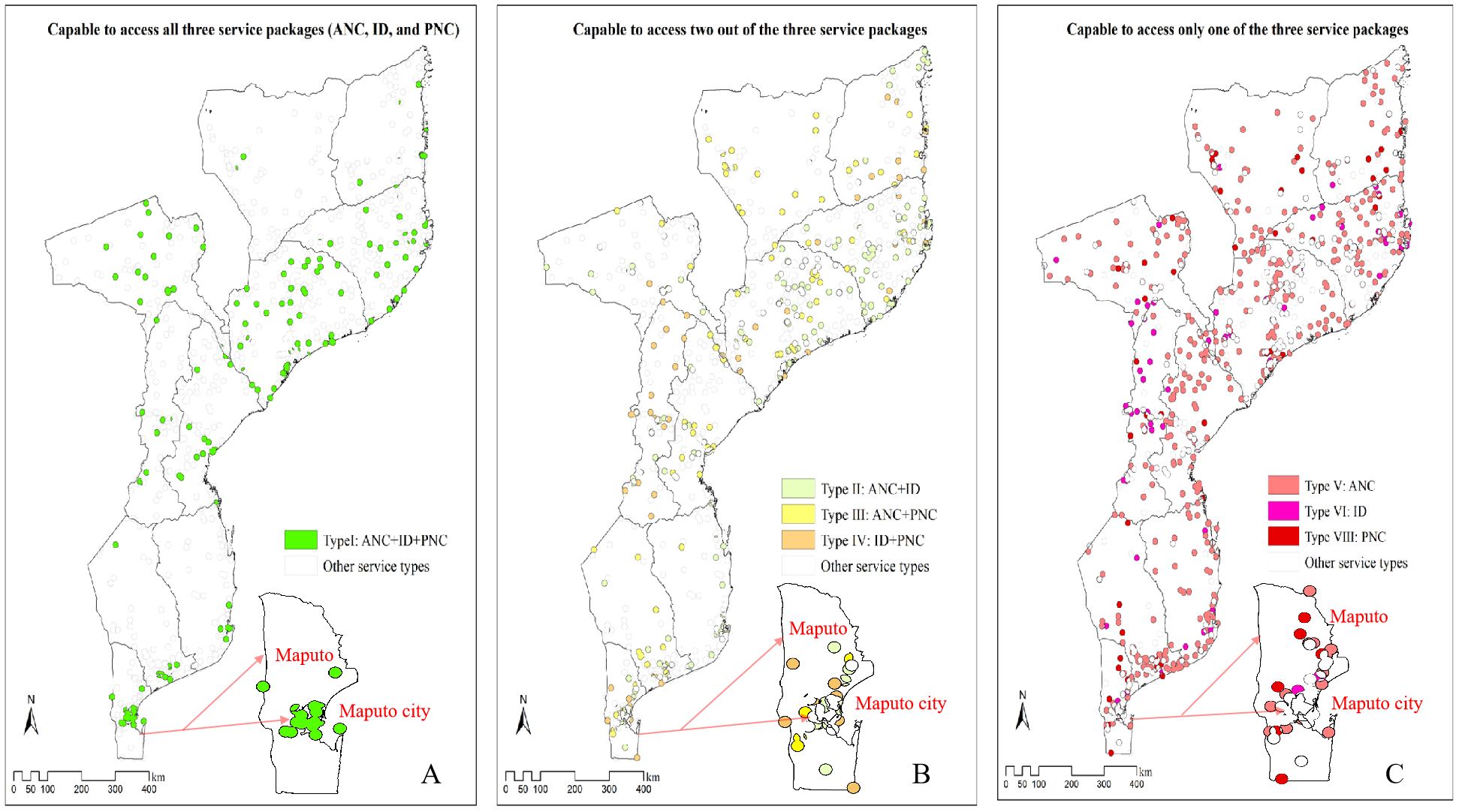

Supplement: Multimedia Appendix 1 [file publichealth_v10i1e49367_app1.docx]
